# Feeder Approach between Trials Is Increased by Uncertainty and Affects Subsequent Choices

**DOI:** 10.1523/ENEURO.0437-17.2017

**Published:** 2018-01-08

**Authors:** Aaron J. Gruber, Rajat Thapa, Sienna H. Randolph

**Affiliations:** Department of Neuroscience, Canadian Centre for Behavioural Neuroscience, University of Lethbridge, 4401 University Dr. W, Lethbridge, AB T1K 3M4, Canada

**Keywords:** exploration, instrumental, lose-shift, Pavlovian, rat, uncertainty.

## Abstract

Animals quickly learn to approach sources of food. Here, we report on a form of approach in which rats made volitional orofacial contact with inactive feeders between trials of a self-paced operant task. This extraneous feeder sampling (EFS) was never reinforced and therefore imposed an opportunity and effort cost. EFS decreased during initial training but persisted thereafter. The relative rate of EFS to operant responding increased with novel changes to the operant chamber, reward devaluation by prefeeding, or lesions to the dorsolateral striatum. We speculate that this may function to increase exploration when the task is uncertain (early in learning or introduction of novel apparatus components), when the opportunity cost is low, or when the learned sensorimotor solution is compromised. Moreover, EFS strongly affected subsequent choices by triggering a lose-shift response away from the sampled feeder, even though it occurred outside of the trial context. This indicates that at least some behaviors occurring between trials impact future behaviors and should be considered in decision-making studies.

## Significance Statement

Collecting resources in natural environments is challenging and often requires balancing the utilization of known sources with the exploration of new ones. Here, we show that at least one behavioral control system in rats promotes contact with feeders when not performing the task, and that this increases with task novelty. This may promote exploration of new ways to attain reward. These feeder contacts influence subsequent choices on the task, apparently by triggering a reward “loss” event affecting other control systems. This interaction among control systems could produce artifacts in laboratory results if not properly controlled, and taking these into account may facilitate analysis of decision-making in freely moving animals.

## Introduction

Optimal reward collection requires the ability to adjust behavior based on past reinforcements and inhibit unproductive actions (Thorndike, 1927). In reinforcement learning theory, the decision-maker’s level of knowledge about the task determines whether an action is productive or not (Sutton and Barto, 1998). If there is no uncertainty because the decision-maker has full knowledge, then all directed actions should exploit the best sources of reward at a rate dictated by need, cost, and risk. Otherwise, the decision-maker should intersperse exploitative actions with some exploratory actions to gain information (Staddon and Motheral, 1978; Kakade and Dayan, 2002; Daw et al., 2006). Exploration allows for discovery of better reward sources or shortcuts to obtain known sources. In practice, humans and animals produce a variety of nonoptimal actions in laboratory tasks (Breland and Breland, 1961; Kahneman and Tversky, 1979; Sugrue et al., 2004; Gruber and Thapa, 2016). Although some can be attributed toward gaining information, much is attributed to a neurobiological failure to execute the optimal action policy or to inhibit underproductive (impulsive) actions (Moeller et al., 2001; Gruber et al., 2010; Bari and Robbins, 2013).

**Figure 1.**
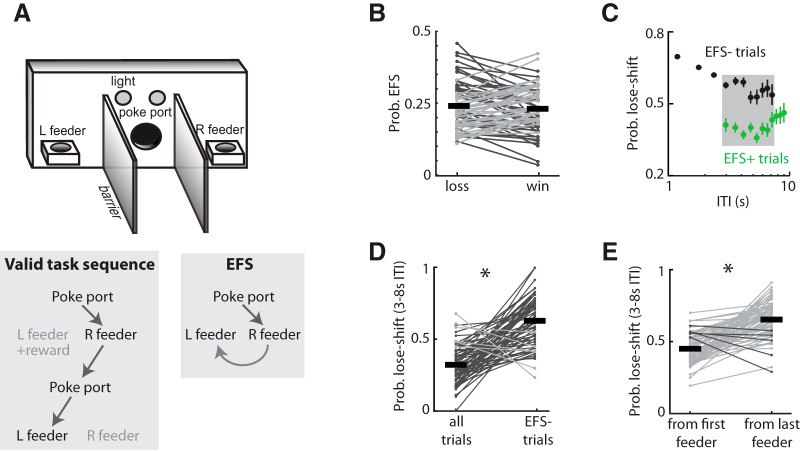
Task apparatus and responding. ***A***, Schematic representation of the behavioral apparatus and examples of operant sequences on the task. Valid sequences consist of a nose poke in the nose-poke port followed by locomotion to one of the two feeders. Rats sometimes chose to locomote from one feeder to the other without committing a nose poke; we term this extraneous feeder sampling (EFS). ***B***, The probability of EFS immediately after reward (win) or reward omission (loss) for each rat (Cohort 1: *n* = 68 for this and subsequent panels), showing that reinforcement does not affect EFS likelihood. ***C***, The probability of lose-shift responding following trials with EFS (green) or no EFS (black) parsed into bins of inter-trial-interval. EFS dramatically reduces lose-shift probability regardless of ITI for the population. ***D***, The within-subject plot of mean lose-shift probability. ***E***, Mean lose-shift probability for each rat computed from either the first feeder chosen after the nose poke or the last feeder chosen before the subsequent nose poke. Nearly all rats appeared to generate lose-shift responses from the last feeder chosen as compared to the first feeder chosen, suggesting that the EFS strongly influences subsequent choice. Error bars indicate SEM, and asterisks (*) indicate group means that were significantly different from the comparison group (*p* < 0.000001).

Impulse control is a composite of processes that span motor, reward/effort, and choice domains (Evenden, 1999; Aron, 2011; Bari and Robbins, 2013). Impulsive actions are often underproductive in laboratory tasks because they lead to suboptimal reward rates, through smaller reward outcomes (Aparicio, 2001; Reynolds et al., 2002) or termination of trials (Carli et al., 1983) or because animals engage in actions that do not lead to reward (Breland and Breland, 1961). Little attention has been given to the influence of such actions on subsequent behavioral choice (Evenden and Robbins, 1984; Williams, 1991). Here we investigate a form of unproductive behavior that we refer to as extraneous feeder sampling (EFS); this occurs when animals ignore task contingencies and choose to make contact with feeders rather than begin the next trial (Fig. 1). This is never reinforced and thus imposes an opportunity cost by consuming time and energy that could otherwise have been spent performing trials to collect reward.

Animals often learn quickly to approach feeders, even when this is not required for reward delivery, such as the goal-tracking response in Pavlovian conditioned approach (Boakes, 1977; Farwell and Ayres, 1979; Robinson and Flagel, 2009). Goal-tracking is reduced by outcome devaluation (Lesaint et al., 2015; Morrison et al., 2015), and the nucleus accumbens core is critical for the expression of Pavlovian conditioned approach (Parkinson et al., 1999; Blaiss and Janak, 2009). We would expect comparable properties of our EFS phenomenon if it involves a Pavlovian component. Moreover, Pavlovian-related learning and memory systems have long been proposed to influence instrumental actions and other behavioral output (Estes and Skinner, 1941; Mowrer, 1947; Rescorla and Solomon, 1967). This likely arises from interactions among distinct behavioral control systems, which in some cases appear to function as opponent processes (Solomon and Corbit, 1974; Boakes, 1977). For instance, pigeons will peck at a stimulus (a Pavlovian-driven action) rather than collect reward via instrumental responding (Williams and Williams, 1969). Moreover, rats approach and engage in operant responding on nearby levers more than distal ones, even if the nearby levers are associated with smaller rewards, require more effort, or impose longer delays to reward (du Hoffmann and Nicola, 2014). This suggests that the brain systems involved in this kind of approach do not use information about relative outcome values and raises the important question of whether approach events can influence future actions, possibly by engaging learning in behavioral control systems that do represent outcomes.

Here, we sought to determine whether EFS affects choice on subsequent trials and whether EFS is related to task uncertainty, impulsivity, or Pavlovian control. Our data suggest that it is related primarily to uncertainty and can affect choices occurring many seconds later involving a different brain structure. This cross-talk between dissociated behavioral control systems is likely important for the study of choice in rodents and possibly other animals.

## Materials and Methods

### Subjects

This study involved 4 cohorts of Long-Evans (LE) rats (*n* = 170 total animals). Cohort 1 consisted of 68 male LE rats obtained from Charles River weighing 450–600 g (postnatal day 94–102) at the time of behavioral testing. All rats were outbred wild-type unless noted otherwise. Cohort 2 consisted of 30 male LE rats (Charles River) weighing 350–450 g (postnatal day 88–106) at the beginning of behavioral testing. Cohort 3 consisted of 16 male and 6 female wild-type LE rats, and 14 male and 15 female LE rats expressing Cre-recombinase under tyrosine hydroxylase (*TH:cre*) born on site and weighing 200–600 g (postnatal day 75–116) at the time of behavioral testing. Cohort 4 consisted of 21 male LE rats obtained from Charles River and weighing 450–600 g (postnatal day 94) at the time of behavioral testing. Housing conditions, training, and testing methods were common to animals from all cohorts. Rats were housed in pairs in a transparent plastic cage with corncob bedding and a section of PVC pipe for enrichment. Access to water was restricted to 1 h per day during behavioral training and testing but was unrestricted otherwise. The vivarium was maintained at 21°C and 12-h light/dark cycle (lights off at 7:30 pm). Experimenters handled the rats daily for 1 wk before the beginning of training. All experimental procedures were approved by the University Animal Welfare Committee and adhere to the guidelines of the Canadian Council on Animal Care.

### Competitive choice task

The competitive choice task (CCT) was used in all experiments. Behavioral training and testing took place in 6 identical custom-built aluminum boxes (26 × 26 cm). Each box contained two cue lights mounted proximally above the nose-poke port and two liquid delivery feeders on either side (Fig. 1*A*). Infrared emitters and sensors in the feeders and central port detected animal entry. After the illumination of the cue lights, the rats poked their snout into the central port to initiate a trial and then responded by locomoting to one of the two feeders. A 13-cm-long aluminum barrier orthogonal to the wall separated each feeder from the central port. This added a choice cost and reduced choice bias originating from body orientation. Control of the behavioral task was automated with a microcontroller (Arduino Mega) receiving commands via serial communication from custom software on a host computer. We reduced acoustic startle from sounds outside of the testing chamber by presenting constant background audio stimuli (local radio station).

All animals were trained on the CCT by gradually shaping components of the task. Initially, there were no barriers between the central port and feeders. Each trial of the task began with the illumination of the two cue lights. At this stage, the animals discovered that every nose-poke port entry and a subsequent entry to either feeder within 15 s resulted in a reward of 60 µL of 10% sucrose solution. Once rats performed 150 trials (typically in the first session), the session was terminated. In the following session, feeder entry was rewarded with a probability of 0.5. Subsequent sessions used the competitive algorithm described below. A barrier separating the nose-poke port and feeders was increased in discrete lengths (4, 8, and 13 cm) over several sessions (typically 4–5). The training was complete when the animals performed at least 150 trials with the 13-cm barrier within the 45-min session over two consecutive days (typically 7–10 training sessions in total).

A computer program served as an opponent for the rats and was implemented as in previous studies (Algorithm 2; Barraclough et al., 2004; Lee et al., 2004; Skelin et al., 2014; Gruber and Thapa, 2016). The algorithm attempts to predict the rat’s next choice by comparing the pattern of choice sequences in the preceding trials (1–4 back) with the choice history of the current session. If any the pattern occurred more likely than chance (computed by the binomial test), the algorithm baited the least likely feeder to be selected on the current trial. If no pattern was detected, the rewarded side was picked randomly. The optimal response policy of the rat is to choose randomly on each trial and disregard reinforcements. The statistical power of the algorithm to detect patterns is initially very weak, and so the rewarded feeder is selected randomly for the first several trials.

### Devaluation

Rats were trained on the CCT and divided into three groups. After all subjects met the training criterion, individuals of each group received free access to a limited amount of the reward (sucrose solution) 20 min before the start of the CCT. The amount of prefeeding was counterbalanced among rats so that an approximately equal number of rats received each of the three prefeeding volumes (0, 5, 10 mL) each testing day. The volume given to each group rotated each of three consecutive days so that each rat had received one of the three levels before behavioral testing.

### Excitotoxic lesions

Surgeries were performed after training was complete in a new group of rats (cohort 4). Rats were then randomly assigned to one of three lesion groups: dorsolateral striatum (DLS, *n* = 7); nucleus accumbens core (NACc, *n* = 7); or control (*n* = 7). All rats received buprenorphine (Alstoe) to mitigate pain 30 min before incision. The animals were anesthetized using 4% isoflurane gas (Benson Medical Industries) in oxygen flowing at 1.0 L/min, and the surgical plane was maintained with 2% isoflurane throughout the surgery. The animals were mounted on a stereotaxic frame (Kopf Instruments), and a midline incision was made to expose the skull. Burr holes were drilled through the skull to allow lowering of infusion cannulas at the following coordinates from bregma [in mm (AP, ML, DV)]: LS (1.6, 3.0, –6.2), (0.8, 3.7, –6.6), (–0.5, 4.5, –6.6); NACc (1.2, 2.1, –7.8). Bilateral lesions of LS and NACc were achieved by microinfusion of quinolinic acid (30 mg/ml in dimethyl sulfoxide, Sigma-Aldrich Canada). A total volume of 0.25 μl quinolinic acid was infused at the rate of 0.175 μl/min in each site using a 30-gauge injection cannula attached to a 10-μl Hamilton syringe via polyethylene tubing (PE-50). The injection cannula was left in place for 2 min after the injection to allow diffusion of the drug. The scalp incision was then closed with sutures. Rats were given subcutaneous injections (0.02 mg/kg) of meloxicam (Boehringer Ingelheim) and monitored for 24 h before returning them to the vivarium. The animals recovered in their home cages (pair housed) for 1 wk before resuming behavioral testing.

At the end of behavioral testing, all subjects received lethal injections of sodium pentobarbital (100 mg/kg i.p.) and were perfused with physiologic saline and 4% paraformaldehyde. The brains were postfixed for 24 h in PFA and then transferred and stored in 30% sucrose in PBS with sodium azide (0.02%) for a minimum of 48 h before sectioning. The brains were sectioned in the coronal plane at 40-μm thickness using an SM2010R freezing microtome (–19°C, Leica). Every second section through the region of interest was wet-mounted on glass microscope slides and later stained with cresyl violet. Images of sections were digitized using a NanoZoomer (Hamamatsu) and evaluated for lesion quality.

### Behavioral analysis

We quantified several behavioral measures in the CCT. EFS was defined as the trials where the animals sampled both feeders after making an entry into the nose-poke port (Fig. 1*A*). The probability of lose-shift was calculated as the probability that the rat would shift feeder choice in the consecutive trial after reward omission. Likewise, the probability of win-stay was calculated as the probability that the rat would repeat the selection of the same feeder on trials immediately after rewarded trials. The number of trials represents the total number of completed trials within a session. Only sessions with >100 trials were included in the analysis, which affected only the analysis of behavior in the rats with lesions to the DLS (1 session of 37 was excluded). The calculation of the percentage of rewarded trials (wins) represents the percentage of all complete trials in which the rat was reinforced with sucrose. Response time measures the time taken to reach the feeder after the exit of nose-poke port, and intertrial interval (ITI) is defined as the time between the first exit of the reward feeder and the next entry into the nose-poke port. Infrared beam break detectors in the feeders were used to detect the number of anticipatory licks during the short hardware-determined delay (typically 200–600 ms) before reward delivery.

Data were analyzed with Matlab (version R2013a; MathWorks) and SPSS (version 21.0; IBM). ANOVA, repeated-measures (RM) analysis of variance ANOVA, and mixed ANOVA were used to assess the significance of lesion on behavioral measures (*p* < 0.05). Where the main effects were statistically significant, a *post hoc* Tukey or Bonferroni test was used to determine which marginal means differed significantly.

## Results

Rats were required to perform a very brief (100-ms) nose poke and then locomote to one of the two adjacent reward feeders for the possibility of receiving sucrose solution as a reward (Fig. 1*A**)*. The optimal behavioral sequence for maximizing the number of rewards on the task is to commit a nose poke in a centrally located port, enter one randomly chosen feeder, and then begin the next trial by committing a nose poke in the port. Locomoting to the alternate feeder (i.e., EFS) without committing the nose poke is never reinforced and has both effort and opportunity costs. We initially suspected that animals would be more likely to approach the alternate feeder after reward omission, compared with reward delivery. However, we found no significant difference in the probability of EFS after a win versus after a loss in well-trained animals in cohort 1 (paired *t* test; *t*_67_ = 0.96, *p* = 0.34; Fig. 1*B*).

We next sought to discern whether EFS affected animals’ choices on subsequent trials. A computer chose the well to be baited on each trial according to each rat’s past actions and reinforcements such that the optimal choice strategy by the rat is a random selection. Nonetheless, most rats tend to engage in the nonoptimal strategy of lose-shift responding above chance levels (i.e., >50% of trials). Previous work has shown that there are several variables that can affect choice on this task. Importantly, the probability of lose-shift responding strongly decays with increasing ITI between the time of reward omission and the start of the next trial on this task (Gruber and Thapa, 2016). This relationship is also present in the current data (black dots in Fig. 1*C*). The EFS behavior increases the ITI because of the additional time it takes to locomote to the alternate feeder before the subsequent nose poke. The ITI distributions for trials after EFS (EFS+) is therefore shifted from that of trials not after EFS (EFS–). We therefore limited the subsequent analysis of lose-shift responding in this cohort to trials with ITI in the range of 3–8 s to ensure sampling from both EFS+ and EFS– trial types throughout the ITI range. The probability of lose-shift is strongly decreased after trials with EFS for all ITI in the test range (green circles in Fig. 1*C*). We hypothesized that this could result from the animals using a lose-shift response from the last feeder sampled in the trial (rather than the first to be sampled). This is strongly supported by two analyses. First, the mean probability of lose-shift for each rat is significantly higher when computed after removing trials following EFS (i.e., mean for the EFS– type) than for the mean computed with all (EFS+ and EFS–) trials (*t*_67_ = 9.1, *p* = 1.00 × 10^–6^ or less; Fig. 1*D*). If the EFS had no effect on subsequent choice, then removing these trials should have had no effect on the mean. Second, the mean lose-shift responding for each rat computed over all trials (EFS+ and EFS–) based on the last feeder visited is much higher than the mean computed from the first feeder visited (*t*_67_ = 10.1, *p* = 1.00 × 10^–6^ or less; Fig. 1*E*). In other words, animals based their lose-shift strategy on the last feeder visited, regardless of whether it was during a trial or not. This suggests that the neural systems involved in this decision-making process mistakenly expected a reward at the second feeder and is consistent with the characterization of lose-shift responding as a “choice reflex” (Gruber and Thapa, 2016). The large effect of EFS on choice motivated us to further investigate its properties and neural basis.

**Figure 2.**
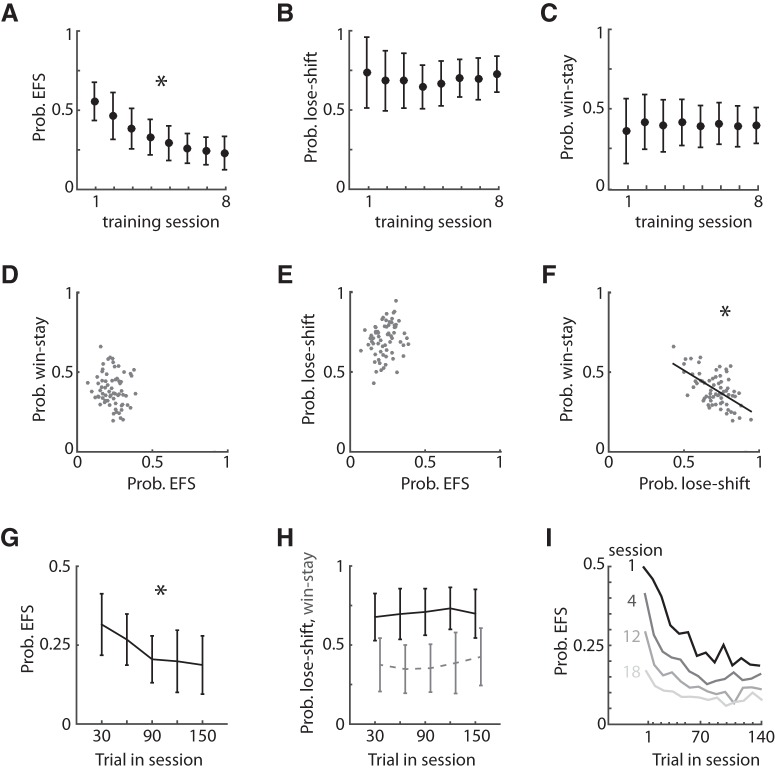
Changes in EFS and win-stay/lose-shift responding within and between sessions. ***A***, The mean probability of EFS decreases over the training sessions (Cohort 1: *n* = 68). ***B***, ***C***, The mean probability of lose-shift or win-stay responding does not change over the training sessions. ***D–F***, Correlations among the probability of EFS, lose-shift, and win-stay responding among rats on the last day of training. The immediate effect of EFS on win-stay and lose-shift measures were minimized by omitting trials following EFS. EFS was uncorrelated with the other response types. ***G***, ***H***, The plot of the probability of EFS, lose-shift, and win-stay (dashed line) for bins of 30 trials within sessions. Only EFS decreased within sessions. ***I***, The plot of EFS probability versus trial bin (10 trials/bin) within each of several sessions of a separate cohort of rats (Cohort 2, *n* = 30), showing that within-session variance of EFS reduces with training. Error bars indicate SEM, and asterisks (*) indicate group means that were significantly different from the comparison group (*p* < 0.000001).

Rats engaged in EFS on nearly 50% of trials in the first few sessions, but this significantly decreased with training (RM-ANOVA, main effects of the session: *F*_7,30_ = 48.95, *p* = 1.00 × 10^–6^; Fig. 2*A*). However, the EFS responses persisted at substantial levels (mean = 0.230 ± 0.106) even after extended training (8 sessions after training was complete). We next sought correlational evidence whether the neural systems promoting EFS are associated with those promoting either win-stay or lose-shift responding, which have distinct properties and neural dependencies (Skelin et al., 2014; Gruber and Thapa, 2016). We excluded all trials after EFS in the subsequent analysis of win-stay and lose-shift responding to avoid the immediate effect of EFS on choice. We examined the session-averaged responses of each rat on the last day o*f* testing (8th session). The rats showed a probability of lose-shift (mean = 0.692 ± 0.020) that was higher than chance levels (*p* = 0.50), consistent with previous reports (Gruber and Thapa, 2016). Lose-shift did not decrease over the training/testing sessions (RM-ANOVA: *F*_1, 36_ = 0.531, *p* = 0.471; Fig. 2*B*). Conversely, the animals showed a lower-than-chance probability of win-stay on the last day of testing (mean = 0.395 ± 0.013), and this again is stable across the training/testing sessions (RM-ANOVA: *F*_7,30_ = 0.427, *p* = 0.877; Fig. 2*C*). We next tested for relationships among these behavioral measures. EFS showed no significant linear correlation with win-stay (*F*_1,67_ = 1.5, *p* = 0.220; *r* ^2^ = 0.02; Fig. 2*D*) or lose-shift (*F*_1,67_ = 3.5, *p* = 0.067; *r* ^2^ = 0.05; Fig. 2*E*) responding, but win-stay was negatively correlated with lose-shift responding (*F*_1,67_ = 34.4, *p* = 1.00 × 10^–6^; *r* ^2^ = 0.34; Fig. 2*F*). This suggests that win-stay and lose-shift are opponent processes or have distinct temporal sensitivities, whereas EFS prevalence is independent of both under normal conditions.

We next wanted to assess whether EFS or the other response variables varied within sessions. EFS responses significantly decreased during the session (*F*_4,64_ = 37.46, *p* = 1.00 × 10^–6^ or less; Fig. 2*G*). In contrast, neither lose-shift nor win-stay responding varied within session (lose-shift: *F*_1,4_ = 7.3, *p* = 0.07; win-stay: *F*_1,4_ = 1.9, *p* = 0.26; Fig. 2*H*). The dissociation of these within-session variances further indicates that EFS is distinct from the neural mechanisms of lose-shift or win-stay responding. The reduction of EFS during the session could be due to changes in either motivation (e.g., thirst) or task uncertainty, which are both expected to decrease as the session progresses. These, however, should diverge with training such that uncertainty should decrease as experience accumulates across sessions, whereas motivation for reward should be relatively invariant among sessions. We therefore examined how EFS decreased within the session as a function of experience (training sessions) in a new group of male LE rats with extended training (cohort 2; *n* = 30). There was a main effect of the training session (*F*_3,84_ = 45.6, *p* = 1.00 × 10^–6^ or less) and of trial in the session (*F*_9,252_ = 27.635, *p* = 1.00 × 10^–6^ or less), as well as a significant trial × session interaction (*F*_27,756_ = 3.34, *p* = 0.001). The within-session decrease became smaller with increased training (Fig. 2*I*) but was still significant at the 18th session (*F*_9,261_ = 4.018, *p* = 1.00 × 10^–6^ or less). These correlational data support the hypothesis that it is task familiarity rather than motivation that drives EFS. We next sought direct evidence for this hypothesis.

**Figure 3.**
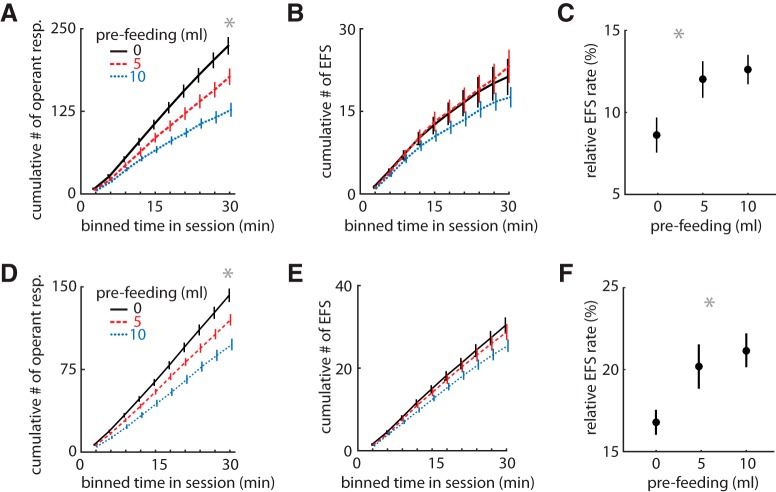
Effect of devaluation on task performance. ***A***, Mean cumulative sum of operant responses (nose poke to feeder) in bins of time within a session (Cohort 2: *n* = 30 rats in ***A–C***). Pre-feeding rats 20 min before the task reduced the number of trials performed. ***B***, The mean cumulative sum of EFS events in the same sessions, which was not reduced by pre-feeding. ***C***, The mean relative rate of EFS/trials for each pre-feeding level, showing an increase with devaluation. ***D–F***, Same plots as above for a new heterogeneous cohort collected by different experimenters (Cohort 3: *n* = 48 in ***D–F***), showing replication of the devaluation effects. Error bars indicate SEM, and asterisks (*) indicate group means that were significantly different from the comparison group (*p* < 0.003).

To discern whether the EFS is promoted by the motivation for the reward, as would be expected by phenomena driven by Pavlovian systems, we conducted a devaluation experiment in cohort 2 after 12 sessions of training. Animals were allowed to drink a fixed amount of liquid sucrose before the task, in a counterbalanced design. This factor should decrease EFS if it is promoted by the motivation for the outcome. Prefeeding decreased the number of trials completed in a volume-dependent manner (RM-ANOVA, main effect: *F*_2,46_ = 35, *p* = 1.00 × 10^–10^; Fig. 3*A*) but had no effect on the number of trials with EFS (*F*_2,46_ = 2.4, *p* = 0.10; Fig. 3*B*). Thus, the relative rate of EFS to operant responses increased with devaluation (RM-ANOVA with Greenhouse–Geisser correction: *F*_1.9,43_ = 6.7, *p* = 0.003; Fig. 3*C*). This was unexpected, and we wanted to test whether this could be an artifact of an unplanned factor within our control. We, therefore, replicated the experiment under conditions of increased variance of originally unplanned factors. The replication was conducted by new investigators (female instead of male), at a different time of year, and with a new heterogeneous group of rats (cohort 3; *n* = 52) that included male LE (*n* = 16), female LE (*n* = 6), transgenic female LE (*n* = 15), and transgenic male LE (*n* = 14) with an inert transgene (see Methods). This cohort was bred in our facility, whereas cohort 2 was shipped from a commercial breeder. Despite these changes, the results were remarkably similar to the first devaluation experiment. Devaluation again decreased trial completion (*F*_1.6,43.8_ = 51.0, *p* = 1.00 × 10^–6^; Fig. 3*D*) but not EFS (*F*_2,50_ = 1.0, *p* = 0.36; Fig. 3*E*), yielding an increased relative rate of EFS (*F*_1.64,41.0_ = 8.0, *p* = 0.002; Fig. 3*F*). Note that the rate of EFS is higher in this group (cohort 3) compared with cohort 2 because they had fewer training sessions before the devaluation. These data provide strong evidence that EFS is a robust phenomenon independent of outcome valuation.

**Figure 4.**
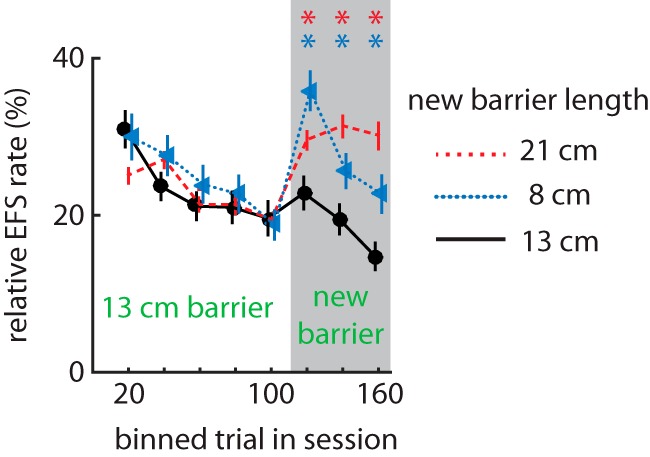
Effect of mid-session change in the barrier on EFS rate. Mean relative EFS rate within a session before and after the barrier was replaced at trial 101 (male subjects from Cohort 3: *n = 16*). Replacing the barrier with either a longer (red dashed line) or shorter (blue dotted line) barrier increased EFS as compared to replacing with the same length barrier (black solid line). Asterisks (*) indicate a significant difference of means by post-hoc analysis *(P* < 0.04).

We next tested whether uncertainty would affect the relative EFS rate. We allowed rats (*n* = 16 male LE wild-type from cohort 3) to perform the task for 100 trials with their customary 13-cm barrier separating the nose-poke port from the feeders. We then took the rats out of the box and replaced the barrier with either a longer one, a shorter one, or one of the same length. Rats were then placed back in the box and allowed to perform an additional 100 trials. The relative EFS rate increased for either novel barrier length compared with the familiar one (RM-ANOVA, time × barrier: *F*_14,294_ = 3.34, *p* = 1.00 × 10^–5^; Fig. 4). These data indicate that EFS is not related to the effort of circumnavigating the barriers, because we would then expect a monotonic length–EFS relationship rather than a parabolic one. These results indicate that a change in the apparatus is sufficient to transiently increase EFS, suggesting that EFS is promoted by uncertainty about the task or apparatus.

**Figure 5.**
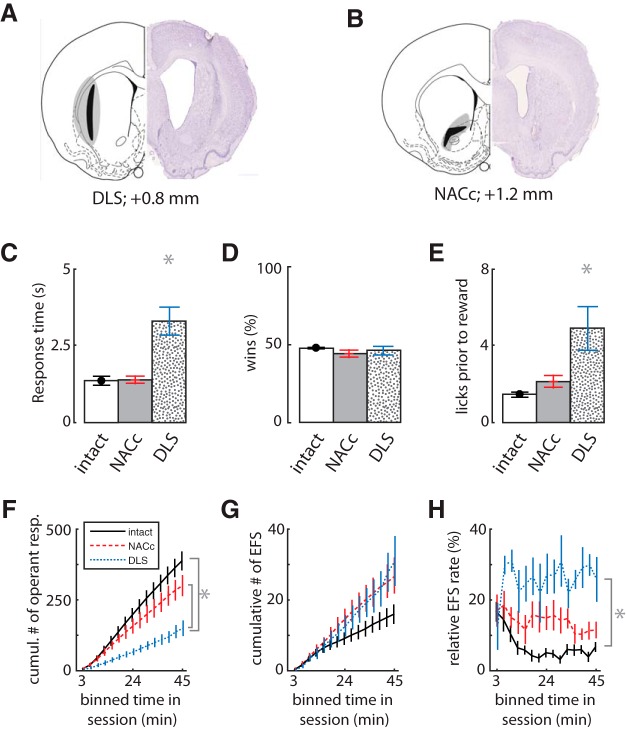
Effects of lesions of the dorsolateral striatum (DLS) or nucleus accumbens core (NACc). ***A***, ***B***, The extent of the excitotoxic striatal lesions in Cohort 4 (*n* = 21). The black and gray shading show minimal and maximal extent of the lesions to the dorsolateral striatum (DLS, *n* = 7) or nucleus accumbens core (NACc, *n* = 7), respectively. ***C***, Mean response times, showing that DLS-lesioned rats made slower responses than the other two groups (*n* = 7 controls). ***D***, The mean percentage of rewarded trials was not affected by lesions. ***E***, Mean number of licks before reward. ***F***, Cumulative sum of completed task trials in bins of time within sessions. ***G***, Cumulative sum of EFS, showing no reduction in lesioned rats relative to controls. ***H***, Relative rate of EFS to operant responses (trials) within sessions, showing a dramatic increase in DLS-lesioned animals. Error bars indicate SEM, and asterisks (*) indicate group means that were significantly different from each other by Tukey’s HSD post-hoc test *(p* < 0.01).

The previous data indicate that EFS is not sensitive to outcome devaluation and therefore not likely directly affected by Pavlovian associations. EFS could instead arise from the inability to suppress motor responses leading to the feeders. Such impulsive actions are typically associated with processing in the sensorimotor regions of the rodent caudate-putamen in the dorsolateral striatum (Graybiel, 1998), which do not show devaluation effects (Balleine et al., 2007). If so, then damage to this region would be expected to reduce the rate of EFS. We tested this by producing bilateral excitotoxic lesions of either the dorsolateral striatum (DLS; *n* = 7) or the nucleus accumbens core (NACc; *n* = 7) and comparing the resultant CCT behavior to control animals (*n* = 7) from the same cohort. The location and extent of the lesions (Fig. 5*A*,*B*) are similar to previous reports from our group and others (Hall et al., 2001; Skelin et al., 2014).

The DLS-lesioned rats had higher response times than controls (*F*_2,16_ = 19.4, *p* = 1.00 × 10^–6^; Fig. 5*C*) but equivalent percentages of rewarded trials compared with controls (*F*_2,16_ = 1.0, *p* = 0.4; Fig. 5*D*). They showed above-normal amounts of licking in the feeder, suggesting no motivational deficit (Fig. 5*E*). The DLS rats had a much lower rate of trial completion than controls (ANOVA main effect: *F*_2,15_ = 16.4, *p* = 2.00 × 10^–4^; Tukey *post hoc* shown in Fig. 5*F*). Their rate of EFS was not statistically different from that of controls, but tended to be higher (*F*_2,15_ = 2.8, *p* = 0.09; Fig. 5*G*). The relative rate of EFS to operant responses was therefore significantly higher in DLS-lesioned animals than controls (*F*_2,15_ = 22.9, *p* = 5.00 × 10^–5^; Fig. 5*H*). The NACc-lesioned rats were not different from controls in either trial completion or EFS (*post hoc* shown in Fig. 5*F–H*). These data indicate that EFS does not depend critically on either striatal region, and further suggests that EFS is not a product of impulsive engagement of habits dependent on the DLS, because DLS lesion did not reduce EFS, and even tended to increase it (Fig. 5*G*).

**Figure 6.**
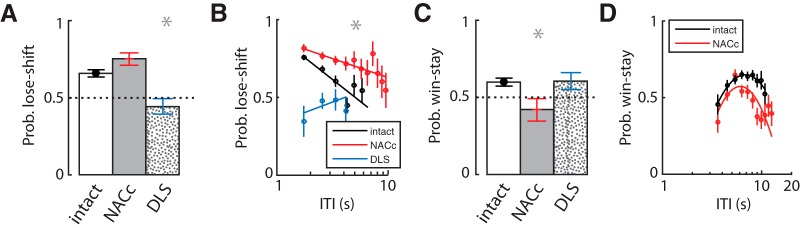
Effect of striatal lesions on lose-shift and win-stay responding. ***A***, The group-averaged probability of lose-shift responding, showing that DLS-lesioned animals decreased lose-shift relative to the other groups, and approached the optimal level in this task (*p* = 0.5). ***B***, The plot of the probability of lose-shift versus the logarithm of the inter-trial-interval for each group. The DLS-lesioned animals show low lose-shift regardless of the ITI. ***C***, The group-averaged probability of win-stay responding. ***D***, The plot of the probability of win-stay versus the logarithm of the inter-trial-interval, showing that animals with NACc lesions have reduced win-stay probabilities regardless of ITI. Error bars indicate SEM, and asterisks (*) indicate group means that were significantly different from each other by Tukey’s HSD post-hoc test *(p* < 0.05).

Further evidence that EFS is independent of these striatal regions comes from the dissociation of lesion effects on EFS from win-stay or lose-shift responding. Consistent with our previous finding (Skelin et al., 2014), the DLS lesion group made significantly fewer lose-shift responses than the control or NACc-lesion groups (*F*_2,16_ = 15.83, *p* = 1.00 × 10^–6^; Fig. 6*A*), and this reduction was irrespective of the ITI (Fig. 6*B*). The DLS-lesioned group had a lose-shift response probability at chance levels for all ITI values. The NACc lesion group, in contrast, showed a higher probability of lose-shift than controls across the range of the ITI (Fig. 6*B*). Furthermore, the large reduction in lose shift in DLS-lesioned animals (compared with controls) is also evident when including EFS+ trials and computing lose-shift from the last feeder sampled (controls = 0.65 ± 0.01; DLS-lesioned = 0.40 ± 0.01; t_10_ = 4.0, *p* = 0.003). The effects of lesion location on win-stay responding had an inverse relationship; the NACc-lesioned group showed a marginally significant reduction in win-stay compared with the other groups (ANOVA main effect: *F*_2,16_ = 3.782, *p* = 0.045; Fig. 6*C*), whereas DLS lesions had no reduction in win-stay (*post hoc* Tukey, *p* = 0.996). This reduction occurred over the range of the ITI (Fig. 6*D*), suggesting that it normally plays a role in suppressing such actions, whereas the NACc lesion group showed a nonsignificant trend for increased EFS compared with controls (*post hoc* Tukey, *p* = 0.061). In sum, lose-shift responding depends on the integrity of the DLS, whereas win-stay depends on the NACc. The number of EFS events was not reduced by either lesion and, in fact, showed a nonsignificant trend to increase in lesioned animals, whereas the ratio of EFS to operant task performance was much higher in DLS-lesioned animals than controls.

## Discussion

Decision-making is a complex process influenced not only by the drive to maximize cumulative reward but also by proximate influences such as the drive to approach feeders, outcome-related cues, and choice reflex tendencies such as lose-shift and win-stay responses. These influences likely involve interactions among multiple brain circuits with unique information-processing capacities (Daw et al., 2005; Balleine and O’Doherty, 2010; Gruber and McDonald, 2012). Here, we have revealed dissociations among regions of the striatum in win-stay, lose-shift, and the suppression of approach to the feeders outside of the normal task sequence (e.g. context). This latter behavior (EFS) was insensitive to reinforcements, but it strongly affected subsequent choice in the task; rats lose-shifted away from the last feeder sampled before the subsequent nose poke, regardless of whether feeder entry was from a choice within the operant task or a consequence of EFS. This is a novel mechanism by which reinforcement-driven task performance could be modulated indirectly by manipulations that affect approach behaviors outside of the task context.

The EFS behavior never fully diminished despite the lack of any positive reinforcement (Fig. 2*I*). EFS occurred in control animals on about a quarter of their trials even after extended training. A similar phenomenon was observed by Boakes (1977) in his study of goal tracking and sign tracking behaviors when reward omission conditions were introduced. The omission contingencies in Boakes (1977) were effective in reducing the frequency of the goal-tracking response, although it rarely eliminated them. Boakes interpreted the failure to diminish responses with reward omission as an indication that the goal-tracking and sign-tracking responses are in competition for behavioral control. We speculate that similar opponent influences result in the persistence of EFS in the CCT. One of these processes drives the instrumental responding and involves the DLS, as evidenced by the reduction in trial completion after lesion of this structure. We have no evidence to suggest what process promotes EFS in the present task.

Although there are no explicit discriminative stimuli predicting reward delivery in our task, we cannot rule out the formation of associative learning involving implicit stimuli. These could involve stimulus-outcome (S-O) or response-outcome (R-O) contingencies when the rat is reinforced at the feeder. Indeed, the use of multiple outcomes and lack of discriminative stimuli promote R-O and/or S-O control (Holland, 2004). It is possible that rats break the operant response into multiple components. If one of these represents entry of the lane to the feeder, it is possible that the R-O of this portion gains strength during training. However, this suggests that the EFS should increase with training, whereas the data reveal that it decreases. Alternately, the feeder could have gained incentive salience because it is the most proximal conditioned stimuli (CS) to the unconditioned stimuli (UCS, i.e., sucrose). Rats, therefore, may be motivated to make an EFS response due to Pavlovian (S-O) attraction to stimuli proximal to the UCS. The main problem with such an interpretation is that the absolute rate of EFS trials was not reduced by the devaluation of the outcome via prefeeding in either of two distinct cohorts. This contrasts the reduction in feeder approach (goal-tracking) by devaluation in other tasks (Lesaint et al., 2015; Morrison et al., 2015), suggesting that these may be distinct phenomena. There is some precedence for this, as rates of magazine entry in some training paradigms are likewise insensitive to devaluation (Killcross and Coutureau, 2003). Note that the EFS behavior requires rats to locomote around a barrier to an unseen feeder, which is not a feature common to past work on this topic. These data suggest that EFS is driven by associations other than R-O or S-O. An alternative mechanism could be stimulus-response (S-R) responding, which is largely unaffected by devaluation and is thought to involve DLS (Graybiel, 1998; Yin and Knowlton, 2004; Yin et al., 2004; Dolan and Dayan, 2013). However, the rate of EFS was not reduced by lesions of the DLS in the present study, suggesting the involvement of some other brain region. An obvious candidate is NACc. Dopamine depletion in this structure drastically reduces engagement in instrumental responding (Nicola, 2010), and NACc neurons encode nearby manipulanda and presumably support approach (Morrison et al., 2015). Moreover, infusion of amphetamine into NACc increased EFS (Wong et al., 2017a), consistent with reports that this manipulation increases Pavlovian conditioned approach (Parkinson et al., 1999; du Hoffmann and Nicola, 2014). It was thus surprising that lesions of NACc in this study did not decrease EFS. Perhaps the extent of lesions was insufficient, or some other brain region can quickly take over the NACc’s contribution to EFS. Nonetheless, this is consistent with proposals that multiple reinforcement learning and memory systems can compete for control of behavior (Dayan et al., 2006).

Is the shuttling between feeders (EFS) simply an error reflecting incomplete mastery of the task contingencies, or does it reveal something about ingrained foraging behaviors in rats? We argue that it is the latter. EFS does not fully extinguish after extensive training and appears to increase at times of less certainty of the task: initial training, the beginning of sessions, and after a switch of the barriers. Its insensitivity to both devaluation and reward outcome (wins/losses) indicates that EFS is not driven by motivation, frustration, or outcome expectation. We therefore speculate that EFS may serve a role in ethological contexts to increase explorative actions. Reinforcement theory indicates that this is a good policy in environments with uncertainty (Sutton and Barto, 1998; Kakade and Dayan, 2002; Sugrue et al., 2004; Daw et al., 2006). We argue that the natural environment involves sufficient variability in such a large state space that animals will always face some level of uncertainty about features pertinent to survival. We speculate that the rodent brain may, therefore, have evolved a system that promotes exploration for foraging, particularly at times of uncertainty or when opportunity costs are low. Moreover, the neural systems promoting exploration may be inhibited as those that promote exploitative actions gain associative strength. This would account for the reduction of EFS with training, and its tendency to increase after striatal lesions in well-trained animals. The within-session decrease in EFS is remarkably similar to the profile of outcome uncertainty in a recent computational model (Daw et al., 2005), thus supporting our interpretation of EFS as being promoted by uncertainty. A postulate of this model is that such uncertainty mediates behavioral control among two reinforcement learning systems – one involving the prefrontal cortex that can use an explicit (model-based) representation of outcome values to predict action outcomes, and another involving the DLS that uses “cached” values (model-free). The rate of responding by the former system is sensitive to devaluation, whereas the latter is not. This model would therefore infer the nose-poke component to be mediated by the model-based system and EFS behavior by the model-free system. We found, however, that lesions of the DLS increased EFS, which conflicts with the model’s prediction. In sum, the dissociation of devaluation effects on the nose-poke and feeder approach elements of task performance suggests that they are mediated by dissociated brain systems.

A striking and unexpected feature of the data is that the feeder approach during the ITI strongly affected subsequent choices on task. We observed that EFS triggered the lose-shift response, suggesting that the reward error signal conveyed to this system treats EFS similar to the operant approach during the task. This lack of context may be explained by the properties of the DLS. We have shown previously (Skelin et al., 2014) and here (Fig. 6*A*,*B*) that lose-shift depends on the lateral striatum, and the dorsal region of this structure is generally not contextually sensitive (McDonald and White, 1993). The ability of EFS to trigger lose-shift responding reveals cross-talk between behavioral control systems that, to our knowledge, has not been previously described. This could be related to proposals that reward prediction error signals in the striatum are “factored” to account for complexity in the world and go on to impact multiple reinforcement learning systems (Lesaint et al., 2014). Furthermore, our results are consistent with the proposal that, in goal-tracking animals, the constant presence of feeders in the testing chamber (often in an inactive state) causes a downward revision in their value, which is then subsequently revised upward on reward delivery during the task (Patitucci et al., 2016). Our finding that EFS engages lose-shift responding supports the postulate of the engagement of a negative reward prediction error on approach outside of the task. This may depend on features of the task. For instance, goal-tracking is linked to the palatability of the reinforcer and sensory associations, suggesting it is not an immutable property of temperament (Lesaint et al., 2015). It remains to be determined whether EFS will similarly depend on reinforcement qualities or sensory stimuli.

EFS is modulated by drugs such as d-amphetamine (Wong et al., 2017a), but not others such as Δ-9-tetrahydrocannabinol (Wong et al., 2017b). Moreover, it appears to be sexually dimorphic in rats and may be subject to modulation by stress, inflammation, or other factors (unpublished observations). Such effects on EFS, and the effect of EFS on subsequent choice, highlight the need to consider actions before trial initialization when analyzing the effects of treatments on decision-making.

## Synthesis

Reviewing Editor: Quentin Pittman, University of Calgary Decisions are customarily a result of the Reviewing Editor and the peer reviewers coming together and discussing their recommendations until a consensus is reached. When revisions are invited, a fact-based synthesis statement explaining their decision and outlining what is needed to prepare a revision will be listed below. Note: If this manuscript was transferred from JNeurosci and a decision was made to accept the manuscript without peer review, a brief statement to this effect will instead be what is listed below.

It is the opinion of the editor that the comments of the reviewers are constructive suggestions for revision and are not criticisms of the data per se. The authors have responded appropriately to the reviews and made the necessary revisions

## References

[B1] Aparicio CF (2001) Overmatching in rats: the barrier choice paradigm. J Exp Anal Behav 75:93–106. 10.1901/jeab.2001.75-93 11256869 PMC1284810

[B2] Aron AR (2011) From reactive to proactive and selective control: developing a richer model for stopping inappropriate responses. Biol Psychiatry 69:e55–e68. 10.1016/j.biopsych.2010.07.024 20932513 PMC3039712

[B3] Balleine BW, O’Doherty JP (2010) Human and rodent homologies in action control: corticostriatal determinants of goal-directed and habitual action. Neuropsychopharmacology 35:48–69. 10.1038/npp.2009.131 19776734 PMC3055420

[B4] Balleine BW, Delgado MR, Hikosaka O (2007) The role of the dorsal striatum in reward and decision-making. J Neurosci 27:8161–8165. 10.1523/JNEUROSCI.1554-07.2007 17670959 PMC6673072

[B5] Bari A, Robbins TW (2013) Inhibition and impulsivity: behavioral and neural basis of response control. Prog Neurobiol 108:44–79. 10.1016/j.pneurobio.2013.06.005 23856628

[B6] Barraclough DJ, Conroy ML, Lee D (2004) Prefrontal cortex and decision making in a mixed-strategy game. Nat Neurosci 7:404–410. 10.1038/nn1209 15004564

[B7] Blaiss CA, Janak PH (2009) The nucleus accumbens core and shell are critical for the expression, but not the consolidation, of Pavlovian conditioned approach. Behav Brain Res 200:22–32. 10.1016/j.bbr.2008.12.024 19159648 PMC4667776

[B8] Boakes RA (1977) Performance on learning to associate a stimulus with positive reinforcement Operant-Pavlovian interactions, 67–97.

[B9] Breland K, Breland M (1961) The misbehavior of organisms. Am Psychol 16:681–684. 10.1037/h0040090

[B10] Carli M, Robbins TW, Evenden JL, Everitt BJ (1983) Effects of lesions to ascending noradrenergic neurones on performance of a 5-choice serial reaction task in rats; implications for theories of dorsal noradrenergic bundle function based on selective attention and arousal. Behav Brain Res 9:361–380. 6639741 10.1016/0166-4328(83)90138-9

[B11] Daw ND, Niv Y, Dayan P (2005) Uncertainty-based competition between prefrontal and dorsolateral striatal systems for behavioral control. Nat Neurosci 8:1704–1711. 10.1038/nn1560 16286932

[B12] Daw ND, O’Doherty JP, Dayan P, Seymour B, Dolan RJ (2006) Cortical substrates for exploratory decisions in humans. Nature 441:876–879. 10.1038/nature04766 16778890 PMC2635947

[B13] Dayan P, Niv Y, Seymour B, Daw ND (2006) The misbehavior of value and the discipline of the will. Neural Netw 19:1153–1160. 10.1016/j.neunet.2006.03.002 16938432

[B14] Dolan RJ, Dayan P (2013) Goals and habits in the brain. Neuron 80:312–325. 10.1016/j.neuron.2013.09.007 24139036 PMC3807793

[B15] du Hoffmann J, Nicola SM (2014) Dopamine invigorates reward seeking by promoting cue-evoked excitation in the nucleus accumbens. J Neurosci 34:14349–14364. 10.1523/JNEUROSCI.3492-14.2014 25339748 PMC4205557

[B16] Estes WK, Skinner BF (1941) Some quantitative properties of anxiety. J Exp Psychol 29:390 10.1037/h0062283

[B17] Evenden J, Robbins T (1984) Win-stay behaviour in the rat. Q J Exp Psychol 36:1–26. 10.1080/14640748408402190

[B18] Evenden JL (1999) Varieties of impulsivity. Psychopharmacology (Berl) 146:348–361. 10550486 10.1007/pl00005481

[B19] Farwell BJ, Ayres JJ (1979) Stimulus-reinforcer and response-reinforcer relations in the control of conditioned appetitive headpoking (“goal tracking”) in rats. Learning Motiv 10:295–312. 10.1016/0023-9690(79)90035-3

[B20] Graybiel AM (1998) The basal ganglia and chunking of action repertoires. Neurobiol Learning Memory 70:119–136. 10.1006/nlme.1998.3843 9753592

[B21] Gruber AJ, McDonald RJ (2012) Context, emotion, and the strategic pursuit of goals: interactions among multiple brain systems controlling motivated behavior. Front Behav Neurosci 6:50. 10.3389/fnbeh.2012.00050 22876225 PMC3411069

[B22] Gruber AJ, Thapa R (2016) The memory trace supporting lose-shift responding decays rapidly after reward omission and is distinct from other learning mechanisms in rats. eNeuro 3:0167-0116.2016. 10.1523/ENEURO.0167-16.2016 PMC5112541 27896312

[B23] Gruber AJ, Calhoon GG, Shusterman I, Schoenbaum G, Roesch MR, O’Donnell P (2010) More is less: a disinhibited prefrontal cortex impairs cognitive flexibility. J Neurosci 30:17102–17110. 10.1523/JNEUROSCI.4623-10.2010 21159980 PMC3073623

[B24] Hall J, Parkinson JA, Connor TM, Dickinson A, Everitt BJ (2001) Involvement of the central nucleus of the amygdala and nucleus accumbens core in mediating Pavlovian influences on instrumental behaviour. Eur J Neurosci 13:1984–1992. 11403692 10.1046/j.0953-816x.2001.01577.x

[B25] Holland PC (2004) Relations between Pavlovian-instrumental transfer and reinforcer devaluation. J Exp Psychol Anim Behav Process 30:104. 10.1037/0097-7403.30.2.104 15078120

[B26] Kahneman D, Tversky A (1979) Prospect theory: an analysis of decision under risk. Econometrica 263–291. 10.2307/1914185

[B27] Kakade S, Dayan P (2002) Dopamine: generalization and bonuses. Neural Netw 15:549–559. 12371511 10.1016/s0893-6080(02)00048-5

[B28] Killcross S, Coutureau E (2003) Coordination of actions and habits in the medial prefrontal cortex of rats. Cereb Cortex 13:400–408. 12631569 10.1093/cercor/13.4.400

[B29] Lee D, Conroy ML, McGreevy BP, Barraclough DJ (2004) Reinforcement learning and decision making in monkeys during a competitive game. Cognitive Brain Res 22:45–58. 10.1016/j.cogbrainres.2004.07.007 15561500

[B30] Lesaint F, Sigaud O, Flagel SB, Robinson TE, Khamassi M (2014) Modelling individual differences in the form of Pavlovian conditioned approach responses: a dual learning systems approach with factored representations. PLoS Comput Biol 10:e1003466. 10.1371/journal.pcbi.1003466 24550719 PMC3923662

[B31] Lesaint F, Sigaud O, Clark JJ, Flagel SB, Khamassi M (2015) Experimental predictions drawn from a computational model of sign-trackers and goal-trackers. J Physiology 109:78–86. 10.1016/j.jphysparis.2014.06.001 24954026 PMC4272685

[B32] McDonald RJ, White NM (1993) A triple dissociation of memory systems: hippocampus, amygdala, and dorsal striatum. Behav Neurosci 107:3. 8447956 10.1037//0735-7044.107.1.3

[B33] Moeller FG, Barratt ES, Dougherty DM, Schmitz JM, Swann AC (2001) Psychiatric aspects of impulsivity. Am J Psychiatry 158:1783–1793. 10.1176/appi.ajp.158.11.1783 11691682

[B34] Morrison SE, Bamkole MA, Nicola SM (2015) Sign tracking, but not goal tracking, is resistant to outcome devaluation. Front Neurosci 9:468 10.3389/fnins.2015.00468 26733783 PMC4679928

[B35] Mowrer O (1947) On the dual nature of learning—a re-interpretation of “conditioning” and “problem-solving”. Harvard Educ Rev 17:102–148.

[B36] Nicola SM (2010) The flexible approach hypothesis: unification of effort and cue-responding hypotheses for the role of nucleus accumbens dopamine in the activation of reward-seeking behavior. J Neurosci 30:16585–16600. 10.1523/JNEUROSCI.3958-10.2010 21147998 PMC3030450

[B37] Parkinson JA, Olmstead MC, Burns LH, Robbins TW, Everitt BJ (1999) Dissociation in effects of lesions of the nucleus accumbens core and shell on appetitive pavlovian approach behavior and the potentiation of conditioned reinforcement and locomotor activity byd-amphetamine. J Neurosci 19:2401–2411. 10066290 10.1523/JNEUROSCI.19-06-02401.1999 PMC6782569

[B38] Patitucci E, Nelson AJD, Dwyer DM, Honey RC (2016) The origins of individual differences in how learning is expressed in rats: a general-process perspective. J Exp Psychol Anim Learn Cogn 42:313–324. 10.1037/xan0000116 27732045 PMC5058353

[B39] Rescorla RA, Solomon RL (1967) Two-process learning theory: relationships between Pavlovian conditioning and instrumental learning. Psychol Rev 74:151. 5342881 10.1037/h0024475

[B40] Reynolds B, de Wit H, Richards J (2002) Delay of gratification and delay discounting in rats. Behav Process 59:157. 12270518 10.1016/s0376-6357(02)00088-8

[B41] Robinson TE, Flagel SB (2009) Dissociating the predictive and incentive motivational properties of reward-related cues through the study of individual differences. Biol Psychiatry 65:869–873. 10.1016/j.biopsych.2008.09.006 18930184 PMC2737368

[B42] Skelin I, Hakstol R, VanOyen J, Mudiayi D, Molina LA, Holec V, Hong NS, Euston DR, McDonald RJ, Gruber AJ (2014) Lesions of dorsal striatum eliminate lose‐switch responding but not mixed‐response strategies in rats. Eur J Neurosci 39:1655–1663. 10.1111/ejn.12518 24602013

[B43] Solomon RL, Corbit JD (1974) An opponent-process theory of motivation. I. Temporal dynamics of affect. Psychol Rev 81:119–145. 4817611 10.1037/h0036128

[B44] Staddon J, Motheral S (1978) On matching and maximizing in operant choice experiments. Psychol Rev 85:436 10.1037/0033-295X.85.5.436

[B45] Sugrue LP, Corrado GS, Newsome WT (2004) Matching behavior and the representation of value in the parietal cortex. Science 304:1782–1787. 10.1126/science.1094765 15205529

[B46] Sutton RS, Barto AG (1998) Reinforcement Learning: An Introduction. MIT Press: Cambridge, MA.

[B47] Thorndike EL (1927) The law of effect. Am J Psychol 39:212–222. 10.2307/1415413

[B48] Williams BA (1991) Choice as a function of local versus molar reinforcement contingencies. J Exp Anal Behav 56:455–473. 10.1901/jeab.1991.56-455 1774539 PMC1323133

[B49] Williams DR, Williams H (1969) Auto‐maintenance in the pigeon: sustained pecking despite contingent non‐reinforcement2. J Exp Anal Behav 12:511–520. 16811370 10.1901/jeab.1969.12-511 PMC1338642

[B50] Wong S, Thapa R, Baenhorst CA, Briggs A, Sawada JA, Gruber AJ (2017a) Opposing effects of acute and chronic d-amphetamine on decision-making in rats. Neuroscience 345:218–228. 27113327 10.1016/j.neuroscience.2016.04.021

[B51] Wong SA, Randolph SH, Ivan VE, Gruber AJ (2017b) Acute Delta-9-tetrahydrocannabinol administration in female rats attenuates immediate responses following losses but not multi-trial reinforcement learning from wins. Behav Brain Res 335:136–144. 10.1016/j.bbr.2017.08.009 28811178

[B52] Yin HH, Knowlton BJ (2004) Contributions of striatal subregions to place and response learning. Learn Mem 11:459–463. 10.1101/lm.81004 15286184 PMC498333

[B53] Yin HH, Knowlton BJ, Balleine BW (2004) Lesions of dorsolateral striatum preserve outcome expectancy but disrupt habit formation in instrumental learning. Eur J Neurosci 19:181–189. 10.1111/j.1460-9568.2004.03095.x 14750976

